# Cryptorchidism: The dog as a study model

**DOI:** 10.3389/fvets.2022.935307

**Published:** 2022-09-13

**Authors:** Norma Hernández-Jardón, Julio César Rojas-Castañeda, Daniel Landero-Huerta, Estefanía Reyes-Cruz, Rafael Reynoso-Robles, María del Lourdes Juárez-Mosqueda, Alfredo Medrano, Fausto Reyes-Delgado, Rosa María Vigueras-Villaseñor

**Affiliations:** ^1^Programa Doctorado en Ciencias de la Producción y de la Salud Animal, Universidad Nacional Autónoma de México, Mexico City, Mexico; ^2^Laboratorio de Biología de la Reproducción, Instituto Nacional de Pediatría, SS, Mexico City, Mexico; ^3^Laboratorio de Morfología Celular y Tisular, Instituto Nacional de Pediatría, SS, Mexico City, Mexico; ^4^Departamento de Morfología, Facultad de Medicina Veterinaria y Zootecnia, Universidad Nacional Autónoma de México, Mexico City, Mexico; ^5^Laboratorio de Reproducción Animal, Facultad de Estudios Superiores Cuautitlán, Universidad Nacional Autónoma de México, Cuautitlán Izcalli, Mexico; ^6^Banfield Pet Hospital-Universidad Nacional Autónoma de México, Mexico City, Mexico

**Keywords:** cryptorchidism, testis, dog, human, seminiferous epithelium, Leydig cell, vimentin

## Abstract

Cryptorchidism (CO) or undescended testicle is an abnormality of male gonadal development that can generate long-term repercussions in men, such as infertility and germ cell neoplasia *in situ* (GCNIS). The origin of these alterations in humans is not completely clear, due to the absence of an animal model with similar testicular development as in humans with CO. This work intends to describe the testicular histological development of dogs with congenital CO, and determine whether the species could adequately serve as a study model for this pathology in humans. The study was carried out with 36 dogs, equally distributed in two groups: healthy control (CTRL) and CO groups. The contralateral testis to the undescended one in CO group of the animals was considered and analyzed. Each group was subdivided in three stages of development: (1) peripubertal stage (6–8 months), (2) young adult (9–48 months) and (3) senile (49–130 months). Histological development, the presence of cells with gonocyte morphology, cell proliferation, testicular lipoperoxidation and hormonal concentrations of testosterone, estradiol, FSH and LH were evaluated and described. In the cryptorchid testes, the first histological alterations appeared from the first stage of development and were maintained until the senile stage. A pronounced testicular lipoperoxidation occurred only in the second stage of development. The histological alterations due to CO were markedly evident in the young adult stage. Testosterone concentrations witnessed a decrease starting from in the second stage and kept on until the last stage. The contralateral testes of the CO animals showed alterations that positioned them between the control and CO testes. Testicular development of dogs with CO is similar to that of humans. The results of the study suggest that this species could serve as a suitable model for the study of CO in humans.

## Introduction

Cryptorchidism (CO) or undescended testis is the most common abnormality of male reproductive development (1). In premature children, the incidence is 1.1–45.3% (2) while in term newborn and 1-year aged males, it is 3–5 and 1.0–1.5% respectively (3–5). The etiology is associated with environmental, anatomical and genetic factors (6). Its importance resides on the long-term effects, since it predisposes patients to the development of infertility and germ cell neoplasia *in situ* (GCNIS) (7, 8). GCNIS is the histological lesion that precedes the development of testicular germ cell tumor (TGCT) (9).

Reported fertility in men who underwent CO and orchidopexy is 89.7% for unilateral CO and 65.3% for bilateral CO (2). Moreover, oligospermia is documented in 43 and 73% of patients with unilateral and bilateral CO respectively (10). It is mentioned that the oxidative stress generated by the high temperature in the suprascrotal area can damage the Sertoli cells function (11). This condition deprives the germ cells of the factors necessary for their differentiation, or their binding proteins (12). Although, it is possible that free radicals directly affect germ cells by inhibiting spermatogenesis (13).

The prevalence of GCNIS is estimated to be 2.9% in men with a history of CO (14). Undifferentiated gonocytes with pluripotent capacity are suggested to be the target of malignancy, and thus the origin of this neoplasm (9, 15). A gonocyte is a cell of embryonic origin that differentiates into a spermatogonia *stem cell* in the last third of gestation period and continues through the first 10 months of postnatal period to give rise to the germ line that will initiate spermatogenesis in the future (16–19).

Knowledge about the etiopathogenesis of GCNIS and infertility in patients with CO is still limited (20). This may be attributed to the current lack of an animal model that show similar testicular development as in human. Inevitably, this situation may have affected the efforts to find alternative ways to reduce the risk of developing testicular cancer and fertility problems in these patients.

The most used animal models for the study of CO in man have been the rat, mouse and rabbit. In these species, CO is induced by the administration of drugs or surgical manipulation, which can generate stress, surgical iatrogenesis and tissue adhesions, thus compromising the results (21). The rat and the mouse have the disadvantages that the period of gonocyte differentiation to spermatogonia (4–10 days of postnatal life) and the so-called “minipuberty” (the first postnatal hours) are short and without quiescence period (22). Therefore, its experimental management implies challenges.

The domestic dog (*Canis familiaris*) presents the advantage of spontaneous CO development, with a frequency of 6.8% (23). In dogs, the CO presentation can be unilateral or bilateral, the retained testis positioned in inguinal or abdominal the right unilateral inguinal region being the most frequent as in humans (23, 24). In addition, CO in the dog increases the risk of presenting testicular neoplasia from 3.6 to 13.6 times (25). Coupled with this, is the likelihood of developing tumors of the germ cells; such as Sertoli-Leydig cells tumor (26), and classic (26) and spermatocytic seminomas (27). Also, the differentiation period of the gonocytes in dogs, as a target of malignancy, extends up to the first 4 months of age (28).

Based on the above exposed points, the objective of this study was to describe the histological development of the canine testis with spontaneous CO, at different stages of development, to determine if this species can be best suited as a study model for this pathology in humans.

## Materials and methods

### Animals

Thirty-six small-breed dogs (≤ 10 kg) were included in the study. The animals were from several private veterinary clinics, UNAM-Banfield Veterinary Hospital and the Office of the Mobile Sterilization Unit for Dogs and Cats in Alvaro Obregon borough, Mexico City, Mexico. Animals that presented infections, chronic degenerative diseases, malnutrition, testicular tumors and problems in the reproductive system unrelated to CO were excluded from the study.

For the purposes of the study, the dogs were divided into two equal groups of 18 animals each, consisting of healthy control (CTRL) and unilateral inguinal CO animals, clinically diagnosed by palpation and ultrasound imaging study. Each group was subdivided with an equal number of animals into three stages of development as follows: (1) peripuberal (6–8 months), representing the age of the spermatogenic onset and also the limit for testicular descent into the scrotum (28). The average age of the CTRL group was 6.42 ± 0.48 months and that of the CO group was 5.6 ± 0.41 months. (2) The age range of young adult animals was (9–48 months), which is the age of sexual maturity (26). The average age of the CTRL group was 41.14 ± 5.14 months and that of the CO group was 29.85 ± 5.35 months; and (3) while the age of senile animals ranged 49–130 months (26, 28), which encompasses the age of possible development of neoplasia (29, 30). The average age of the CTRL group was 102.85 ± 6.85 months and that of the CO group was 122.57 ± 11.04 months. In addition, in the CO group, the contralateral testicle (CONTRA) to the cryptorchid one, which was always in scrotal position, was analyzed. The testicular samples were obtained at orquiectomy; from each gonad, three samples of testicular tissue, ~0.25 cm^3^ each, from the mid-portion, opposite to the testicular mediastinum were obtained for morphological analysis, two samples of ~0.75 cm^3^ for immunohistochemistry and two of ~0.75 cm^3^ for the determination of lipoperoxidation. In addition, peripheral blood samples were obtained and used to hormonal determination.

### Morphological analysis

The histological alterations induced by canine spontaneous CO condition were used to compare for resemblance with the histological pattern of human CO development.

The evaluation was performed by fixing testicular tissue samples in Karnovsky solution for 24 h, postfixed in 1% OsO_4_, dehydrated in gradual ethanol solutions and embedded in EPON 812 (Ted Pella, INC. CA, USA). Semi-thin sections of 1 μm thickness were cut using an Ultracut UCT ultramicrotome (Leica, Vienna, Austria) and stained with 0.5% toluidine blue. Histological analysis of the seminiferous tubules was performed using a light microscope (BX 51, Olympus, Tokyo, Japan). From each tissue sample, a slide with 5 tissue sections was obtained. Therefore, 5 cross-sectioned tubules were evaluated, resulting in a total of 15 tubules per animal. The area of the seminiferous epithelium was determined by subtracting the internal area from the external area. The cellular area of at least 15 Leydig cells per animal was also determined using an image analysis system (Image-Pro Plus 6.2. Media Cybernetics, INC. MD, USA). Leydig cells were evaluated using the 100x objective lens and identified by their central location in the interstitial space, its polygonal shape of round nucleus, with an evident nucleolus. The maturation index (MI) or Johnsen index (31) and the histopathological index (HI) were determined (32).

To confirm the presence of gonocytes and cellular alterations, the testicular tissue was sectioned at 60–70 nm thickness. Sections were counterstained with uranyl acetate and lead citrate and analyzed with an electron microscope (JEM-1011, JEOL, Osaka, Japan). The presence of gonocytes was determined through their ultramorphological characteristics, among which are large round cells with the presence of cytoplasmic extensions in some cases, prominent nucleus and nucleolus, and mitochondria peripheral to the nucleus.

### Determination of cell proliferation and the functional status of Sertoli cells

Immunoreactivity to proliferating cell nuclear antigen (PCNA) and to vimentin antigen were evaluated using immunohistochemical techniques to determine if the spontaneous CO in the dogs presents cell hypoplasia due to low cell proliferation and alterations in the functional status of Sertoli cells.

The determination was carried out by fixing the two testicular samples per gonad in 4% paraformaldehyde for 2 h. Later, these were dehydrated, clarified and embedded in paraffin. Sections of 4 μm thickness were cut and mounted on slides with poly-L- lysine (Sigma-Aldrich, St. Louis, MO, USA). Tissue sections were deparaffinized with xylene and hydrated with graded ethanol solutions. Subsequently, tissue sections were exposed to sodium citrate (pH 7.6) for 5 min in a microwave oven at 800 W. Afterwards, sections were outlined with PAP pen for immunostaining (Dako, Carpinteria, CA, USA). The tissue sections were then treated with 3% hydrogen peroxide for 10 min. Subsequently, they were placed in 0.1% Tween 20 (Sigma-Aldrich) in phosphate buffered saline (PBS, pH 7.4) for 10 min and blocked with 1% bovine serum albumin in PBS for 2 h. Thereafter, they were incubated overnight at room temperature with mouse monoclonal primary antibody against PCNA (sc-56 B1717, Santa Cruz, Biotechnology, CA, USA) and the primary monoclonal antibody against vimentin (sc-6260, Santa Cruz Biotechnology) both at a dilution of 1:500. This was followed by another incubation with biotinylated anti-mouse IgG (Invitrogen, Carlsbad, CA,USA) at a dilution of 1:100 for 1 h. Finally, the samples were exposed to the streptavidin-horseradish peroxidase conjugate for 1 h (Vector Laboratories, Peterborough, UK). To visualize the immunoreaction, tissue sections were incubated in a peroxidase substrate solution containing 1.6 ml distilled H_2_O, 20 μl 10x substrate buffer and 40 μl 50x diaminobenzidine (chromogen) (Santa Cruz Biotechnology) and 1% H_2_O_2_ (Merck, Darmstadt, Germany) in methanol for 30 min. Subsequently, sections were counterstained with hematoxylin, dehydrated through a graded ethanol series and washed with xylene. All dilutions and washes between steps were done with PBS. The number of immunoreactive cells per transversely cut seminiferous tube was determined. 5 seminiferous tubes per section were evaluated, making a total of 10 tubes evaluated per animal. The distribution pattern of immunoreactivity to vimentin in the cytoplasm of Sertoli cells was also determined. In addition, the Masson trichrome technique was performed on one slide of each animal to show the collagen fibers.

### Determination of lipoperoxidation

Malondialdehyde, the final product of testicular lipoperoxidation, was determined using TBARS technique with the objective to know if the spontaneous CO in the dogs presents an increase in lipoperoxidation as an indirect way of oxidative damage.

For this purpose, 1 ml aliquot containing homogenized testicular tissue was added to 2 ml of thiobarbituric acid reagent (TBA; MP, Illkirch, France) (0.375 g of TBA + 15 g of trichloroacetic acid + 2.5 ml of HCl diluted in 100 ml distilled water). The resulting solution (3 ml total volume) was heated in a boiling water bath for 30 min. Samples were cooled on ice and centrifuged at 3,000 g for 15 min. The absorbance was measured in the supernatants by spectrophotometry at 532 nm. The concentrations of substances reactive with thiobarbituric acid were calculated by interpolation on a standard curve of periodic oxidation of malondialdehyde. The final result was expressed as nmol of substances reactive to thiobarbituric acid per mg of protein. Protein content in testicular tissue samples was measured with Folin's phenol reagent (33). Lipid peroxidation results were corrected for protein content in each sample and expressed as pg MDA/mg protein.

### Hormonal determination

Peripheral blood (5 ml) was performed at 9:00 AM to avoid circadian fluctuations and collected in sterile BD vacutainer tubes for serum extraction and centrifuged at 3500 rpm for 10 min. The serum obtained was stored at −70°C until analysis. The determination of testosterone, estradiol, LH, and FSH was performed for each individual without replicates and by automated chemiluminescent microparticle immunoassay, using the Architect R 2nd generation (Abbott Diagnostics, Abbott Park, IL, USA), with strict adherence to the manufacturers' instructions. The detection limits and the intra- and inter-assay coefficients of variation for each hormone, respectively, were as follows: testosterone 0.01 ng/ml, 3.10 and 3.60%; estradiol 10.00 pg/ml, 2.98 and 3.60%; LH 0.50 mIU/ml, 2.07 and 3.74%; FSH 0.05 mIU/mL, 2.99 and 3.80%.

### Statistical analysis

The data were expressed as the mean ± SEM. A parametric ANOVA test, followed by a multiple comparison using the Tukey test (seminiferous epithelial area, MI, HI, proliferation index, and hormone concentrations) and non-parametric Kruskal–Wallis (lipoperoxidation) test were used for statistical analysis with *P* values < 0.05 considered statistically significant.

## Results

### Stage 1: Peripuberal

**Figure 1:**
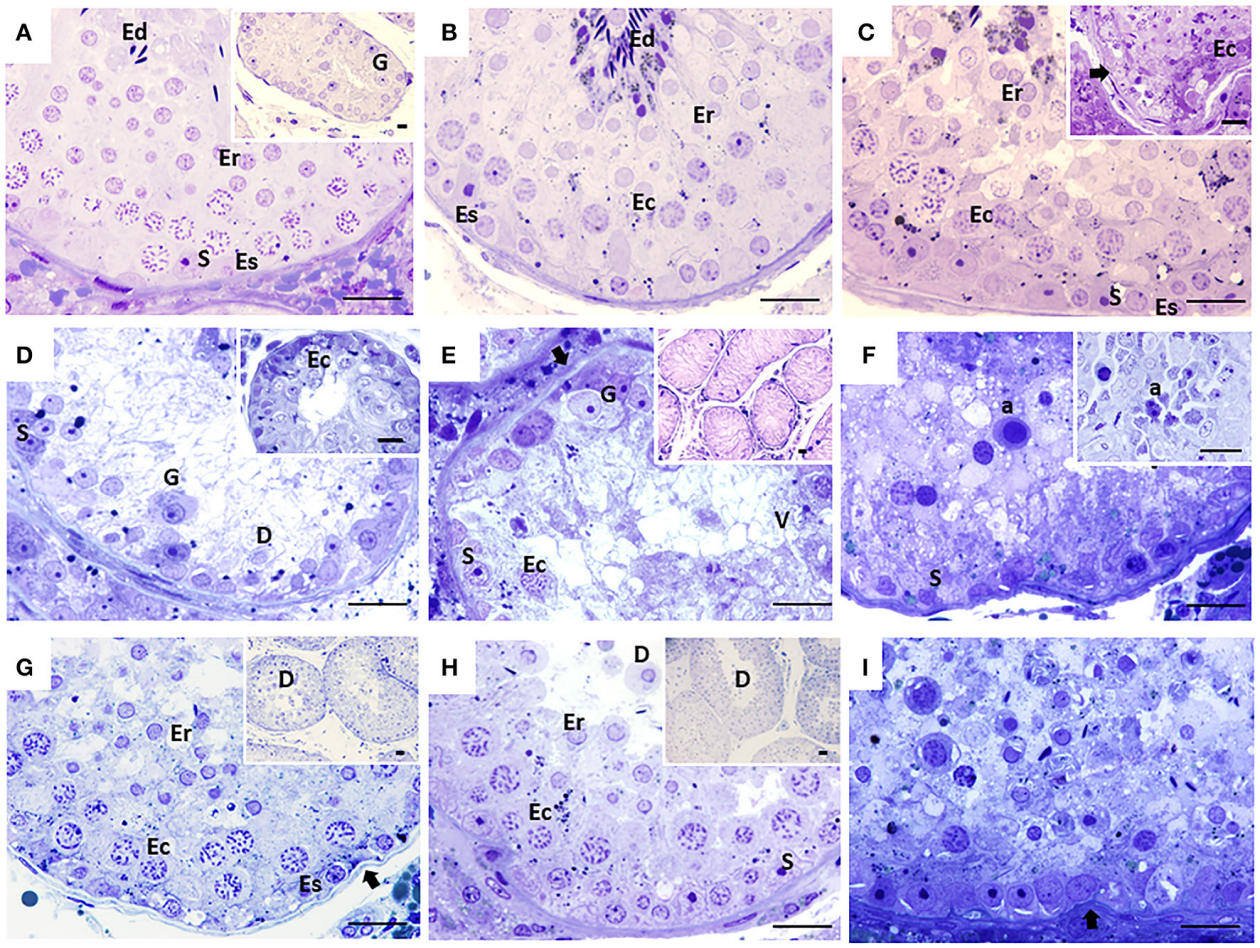
Seminiferous tubules of dogs from the control (CTRL) **(A–C)**, cryptorchid (CO) **(D–F)** and contralateral (CONTRA) **(G–I)** groups at peripubertal (first column); young adult (second column) and senile (third column) stages. In CTRL dogs, complete spermatogenesis is observed, although gonocytes were still observed in the peripubertal stage **(A)**. In the senile stage **(C)**, some specimens showed histological alterations such as thickening and folding in the basement membrane (arrow) and even complete disorganization of the seminiferous epithelium (**C** insert). In animals with CO in the peripubertal stage, different degrees of maturation of the epithelium are observed, from the presence of gonocytes to spermatocytes (**D** insert). Histological alterations become evident from the young adult stage **(E)**, which continued until senile **(F)** with vacuolization (V), desquamation (D), folding of the basement membrane (arrow), presence of atypical cells **(F)**, cellular degeneration and arrest of spermatogenesis, as well as Sertoli-cell-only syndrome (**E** insert). In the contralateral testes, the degree of histological damage was not as severe as in the cryptorchid ones, presenting alterations such as hypoplasia and cellular degeneration, thickening and folding of the basement membrane (arrow) with cellular desquamation (D). The CONTRA group images inserts show a panoramic view of the tubes. Gonocyte (G), Spermatogonia (Es) spermatocyte (Ec), Round spermatid (Er), elongated spermatid (Ed), atypical cells (a), Sertoli cell nuclei (S). Toluidine Blue. Bar scale: 20μm.

**Figure 2:**
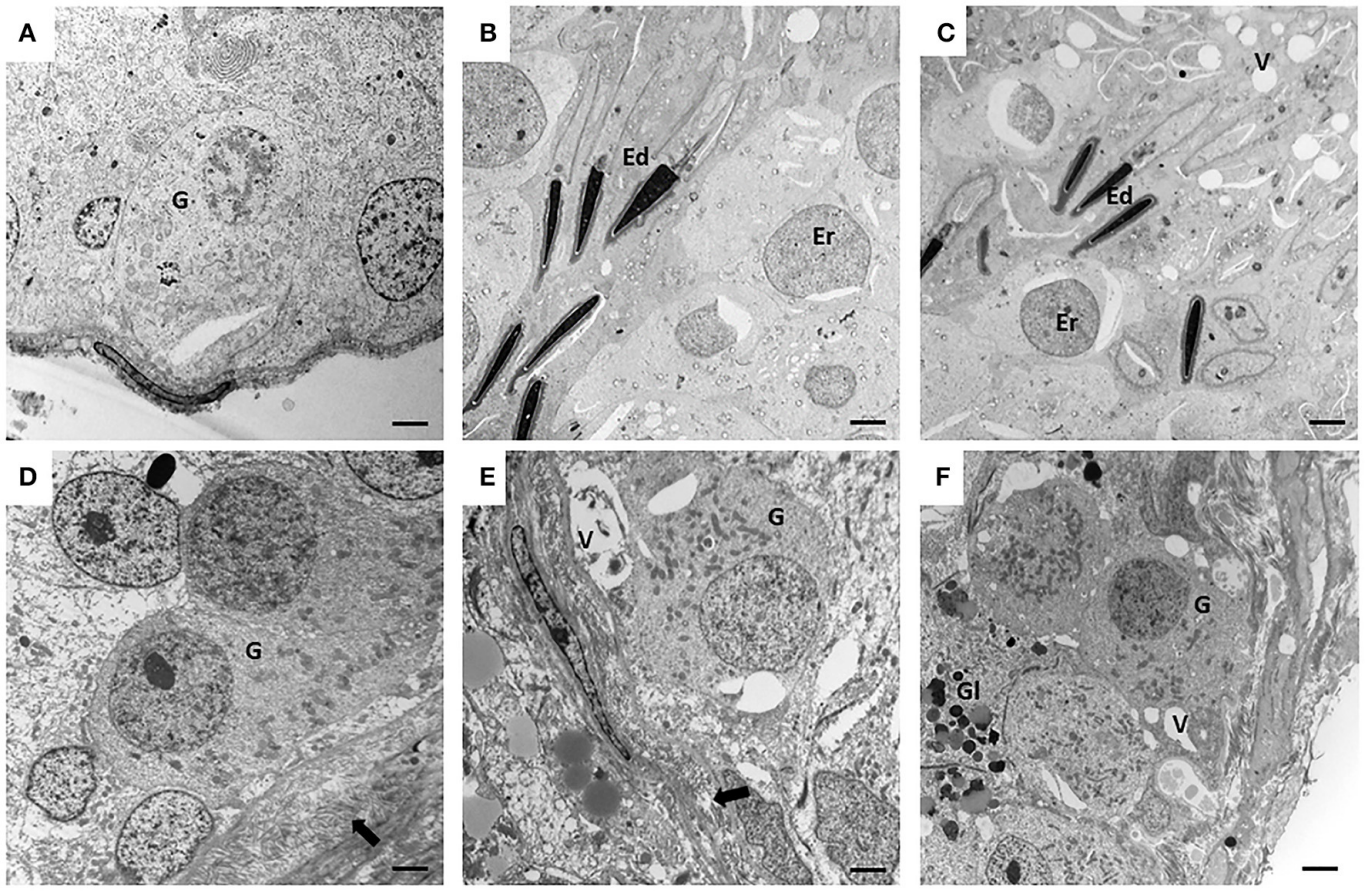
Electron microscopy (EM) of canine testes from the control groups (CTRL) **(A–C)** and from cryptorchidism (CO) **(D–F)**. It is observed in the peripubertal stage, the presence of gonocytes in migration phase **(A)** and with small pseudopodia touching the basement membrane **(D)**. In the young adult stage of the second row, a clear spermatogenesis is observed in the CTRL testis **(B)**, unlike the CO testis, which shows the presence of a gonocyte-like cell with thickening of the basement membrane **(E)**. In the senile stage, the CTRL testes still preserve spermatogenesis **(C)** with disorganization of the epithelium, and cells morphologically similar to gonocytes **(F)** were observed in the CO. Gonocyte (G), round spermatid (Er), elongated spermatid (Ed), basement membrane (arrow), vacuoles (V), glycogen (Gl). Electron microscopy. Bar scale: 2μm.

The CTRL group animals presented different degrees of development of spermatogenesis. The 16.6% of the animals (1/6) presented few gonocytes (verified with electron microscopy), spermatogonia and spermatocytes. In other specimens, it was possible to observe round and elongated spermatids. Slight histological alterations such as scaling were observed (Figure 1A). The ultrastructure showed completely healthy Sertoli and germ cells (Figure 2A).

**Figure 3:**
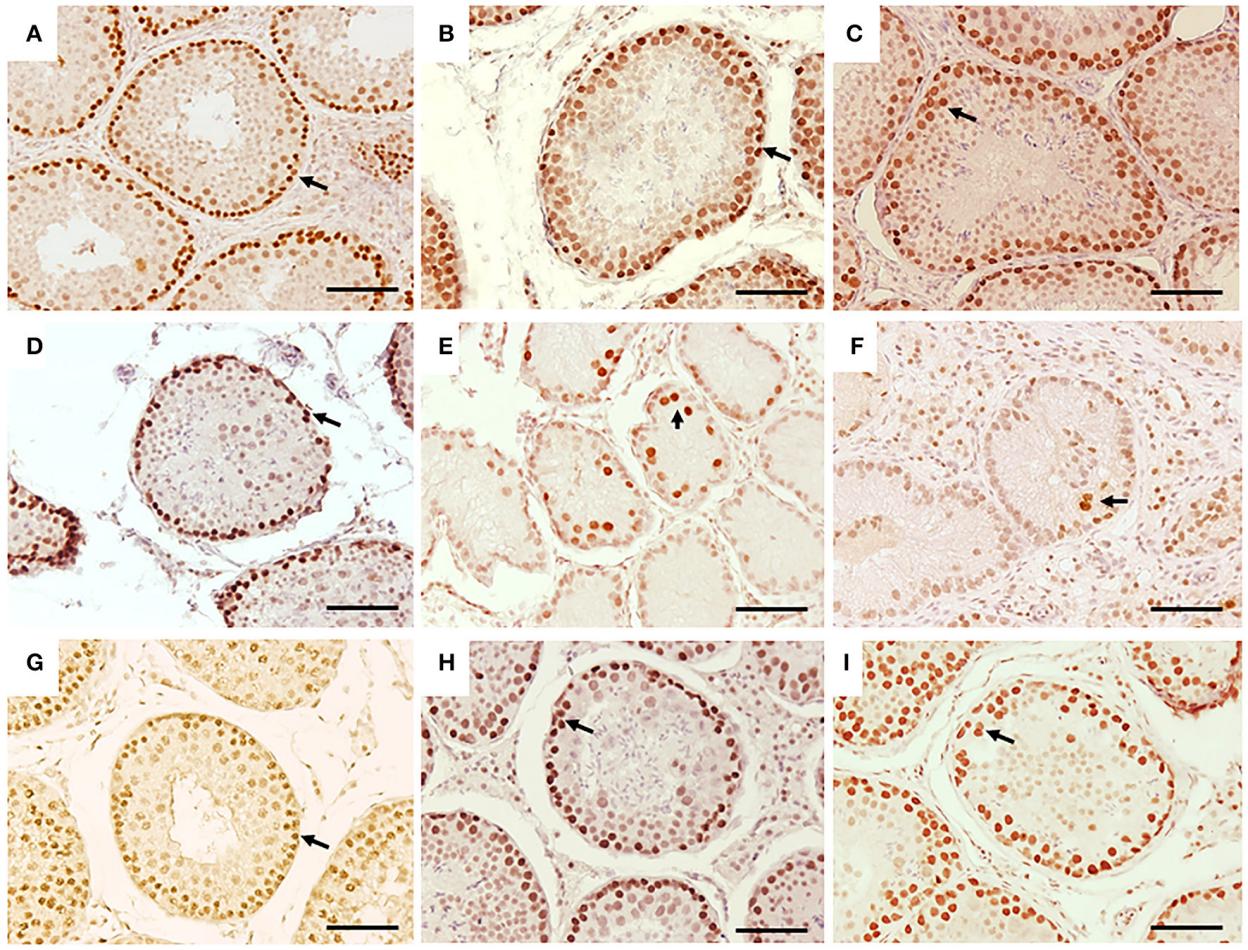
Immunohistochemistry for PCNA in seminiferous tubes of dogs from the control group (CTRL) **(A–C)**; from cryptorchidism (CO) **(D–F)** and contralateral (CONTRA) **(G–I)** in the peripubertal stages (first column); young adult (second column) and senile (third column). A marked immunoreactivity (arrow) is observed in the testes of the CTRL animals unlike the CO, mainly in the young adult stages. The testes of the CONTRA group show an immunoreactivity that is neither as marked as in the CTRL nor as weak as in the CO. Bar scale: 50 μm.

Fifty percent (3/6) of the animals of CO group, presented cells with gonocyte morphology (Figure 1D). Spermatogenesis did not develop beyond spermatocytes (Figure 1D) and slight histological alterations, such as tubular hypoplasia and cellular degeneration, mainly in spermatocytes, were observed in their seminiferous tubules. The animals of the CO group presented a significant reduction in the maturation index (MI) of 2.57 times as well as in cell proliferation index 4.1 times when compared with the CTRL group (Table 1; Figures 1D, 3A,D). However, histopathological index (HI) and seminiferous epithelial area did not show differences between both groups (*p* > 0.05). The result of TBARS technique did not show significant difference (*p* > 0.05) between both groups, although there was a tendency for greater lipoperoxidation in the CO group (2.07 times more. The analysis of the hormones show that T concentrations experienced a downward trend in the CO group when compared with the CTRL group, nevertheless, this was not significant (Table 2). The same trend was observed with the rest of the hormones (Table 2).

**Table 1:** Parameters evaluated in the studied groups at different stages of development of the dogs.

**Development period**	**Group**	**Seminiferous epithelial area** **(μm^2^ X10^2^)**	**Histopathologic index (HI)**	**Maturation index (MI)**	**Proliferation Index (PI)**	**Lipoperoxidation** **(pg MDA/mg of protein**	**Leydig cells area**
Peripuberal	CTRL	177.46 ± 39.42	4.76 ± 0.62	8.11 ± 0.80	96.57 ± 10.97	0.14 ± 0.01	165.51 ± 13.05
	CO	84.78 ± 19.17	10.20 ± 1.45	3.15 ± 0.15^a^	24.05 ± 3.85^a^	0.29 ± 0.11	127.78 ± 21.59
	CONTRA	137.55 ± 42.82	10.85 ± 0.94	4.00 ± 0.40	29.55 ± 7.88	0.16 ± 0.02	128.98 ± 16.95
	*p*-value	>0.05	>0.05	0.013	0.030	>0.05	>0.05
Young adult	CTRL	369.27 ± 26.99	3.07 ± 0.33	9.35 ± 0.29	115.83 ± 11.53	0.13 ± 0.00	205.29 ± 14.87
	CO	100.56 ± 17.73^a^	13.45 ± 1.12^a^	3.46 ± 0.77^a^	25.56 ± 7.36^a^	0.40 ± 0.14^a^	122.94 ± 14.27^a^
	CONTRA	172.96 ± 43.67	6.56 ± 2.90	6.16 ± 1.70	102.20 ± 32.49	0.39 ± 0.18	198.15 ± 15.46^c^
	*p*-value	0.030	0.008	0.0001	0.002	0.0001	<0.037
Senile	CTRL	494.19 ± 128.40	3.42 ± 0.45	9.19 ± 0.22	122.00 ± 13.78	0.17 ± 0.01	197.44 ± 10.84
	CO	114.72 ± 13.41^a^	16.72 ± 2.22^a^	2.93 ± 0.70^a^	43.28 ± 10.13^a^	0.22 ± 0.03	123.10 ± 16.32^a^
	CONTRA	156.09 ± 25.74^b^	13.35 ± 6.20^b^	7.20 ± 1.73^c^	57.68 ± 22.30	0.21 ± 0.02	180.70 ± 13.08
	*p*-value	<0.017	<0.040	<0.03	0.002	>0.05	0.036

The results are expressed as mean ± standard error. Control (CTRL), with cryptorchidism (CO) and contralateral (CONTRA) to the undescended testicle.

^a^ p < 0.05 CTRL vs. CO of the same period of development.

^b^ p < 0.05 CTRL vs. CONTRA of the same period of development.

^c^ p < 0.05 CO vs. CONTRA the same development period.

**Table 2:** Hormone concentrations in the control and cryptorchid groups at different developmental stages in the dog.

**Development period**	**Group**	**Testosterone (ng/ml)**	**Estradiol (pg/mL)**	**FSH (mUI/mL)**	**LH** **(mUI/mL)**
Peripuberal	CTRL	3.85 ± 1.39	10.86 ± 0.70	0.05 ± 0.00	0.50 ± 0.01
	CO *p*-value	0.87 ± 0.36 >0.05	10.50 ± 0.28 >0.05	0.06 ± 0.02 >0.05	0.50 ± 0.03 >0.05
Young adult	CTRL	5.97 ± 1.01	13.75 ± 1.40	0.05 ± 0.00	0.50 ± 0.05
	CO *p*-value	1.22 ± 0.67^a^ 0.044	11.00 ± 0.40 >0.05	0.05 ± 0.00 >0.05	0.50 ± 0.06 >0.05
Senile	CTRL	9.19 ± 1.34	13.83 ± 0.47	0.05 ± 0.00	0.50 ± 0.01
	CO *p*-value	1.41 ± 0.47^a^ 0.0001	12.00 ± 1.18 >0.05	0.06 ± 0.008 >0.05	0.50 ± 0.01 >0.05

The results are expressed as mean ± standard error. Control (CTRL) and with cryptorchidism (CO).

^a^p < 0.05 CTRL vs. CO of the same period of development.

In the CO group, the contralateral testes showed seminiferous tubes with different stages of development, which go from gonocytes to round spermatids (Figure 1G). There were no differences in the variables studied when compared to the CTRL and CO groups (*p* > 0.05) (Table 1; Figure 3G).

**Figure 4:**
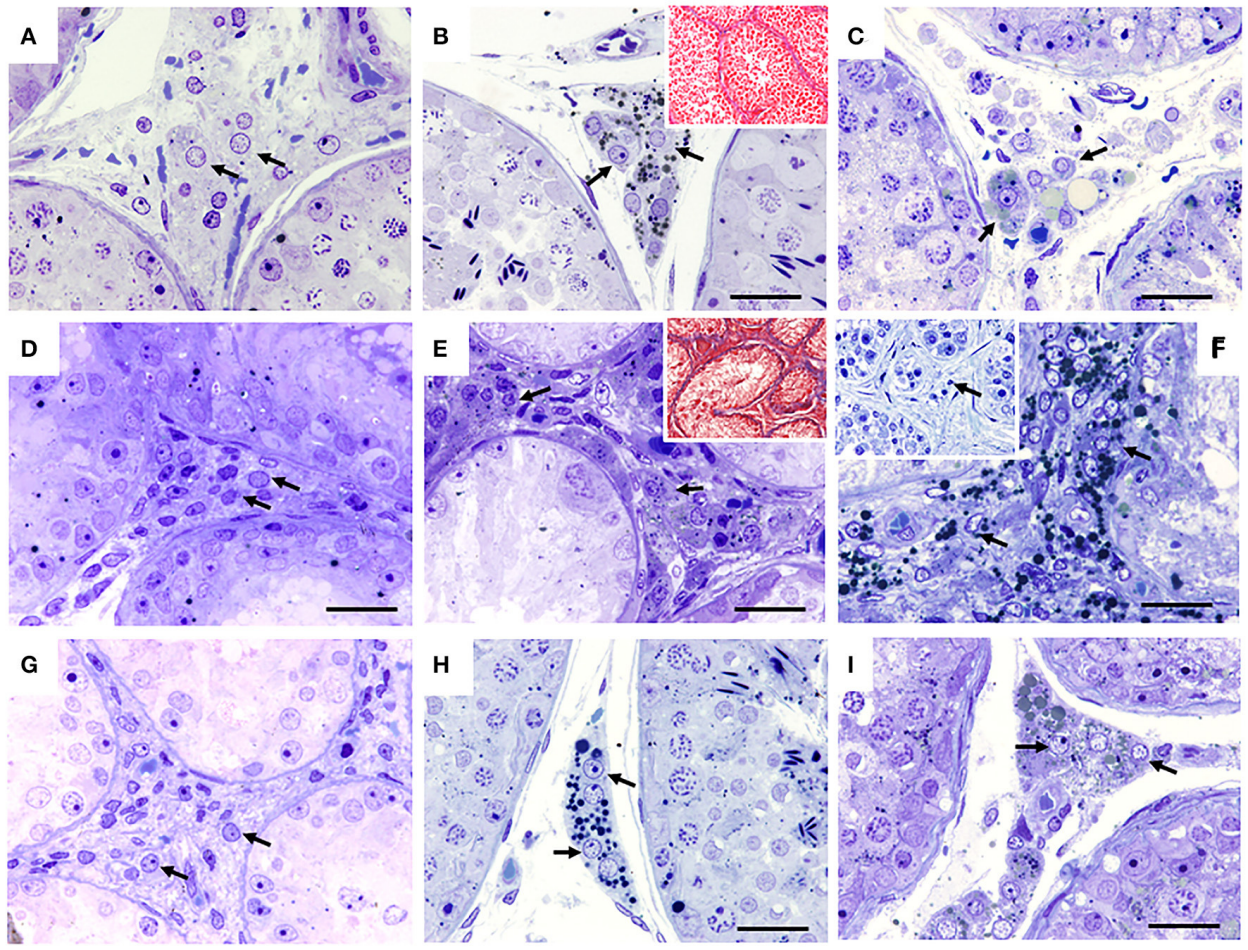
Seminiferous tubules of dogs from the control (CTRL) **(A–C)**, cryptorchid (CO) **(D–F)** and contralateral (CONTRA) **(G–I)** groups at peripubertal (first column); young adult (second column) and senile (third column) stages. The interstitial spaces with their characteristic Leydig cells (arrow) are observed. The young adult and senile stage CO group showed small Leydig cell hyperplasia, when compared with the other groups. In some cases in the CO group in the senile stage, completely degenerated seminiferous tubes can be seen with some degenerating or absent Leydig cells (**F** insert). Toluidine Blue, except **(B,C)** Masson trichrome technique. Bar scale: 50 μm.

In the CTRL, CO groups and the contralateral testes, Leydig cells were observed occupying the central spaces of the tubular interstitium. They were characterized by their polygonal shape of central nuclei that may present lipid droplets (Figures 4A,D,G).

### Stage 2: Young adult

The CTRL group complete spermatogenesis with slight histological alterations, such as vacuolization and desquamation, in the seminiferous epithelium were observed. The seminiferous tubules showed marked proliferation (Figure 3B), however, there was absence of gonocytes in all of them (Figures 1B, 2B).

In the CO group, a marked hypoplasia of germ cells was observed in the seminiferous epithelium, vacuolization, desquamation, folding and thickening of the basement membrane with cell degeneration and cytoplasmic extensions of Sertoli cells directed toward the center of the tubules (Figure 1E). Spermatogenesis did not continue beyond the spermatocytes, and in some specimens, no germ cells were observed. Sertoli-cell-only syndrome was identified in 50% of the animals (3/6) (Figure 1E insert). In addition, they depicted the presence of cells with gonocyte morphology in 16.6% of the specimens (1/6) (Figures 1E, 2E). The animals of the CO group presented a significant reduction of 3.67 times in the area of the seminiferous epithelium, de 2.70 times in MI and de 4.53 times in PI (Figure 3E) with a significant increase in HI of 4.38 times when compared with the CTRL group (Table 1).

At this age, there was a reduction of 4.89 times in testosterone concentrations in the CO group, without significant changes in estradiol, LH and FSH concentrations (*p* > 0.05, Table 2).

The TBARS technique showed a significant increase (*p* < 0.05) of 3.07 times in lipid peroxidation in CO testes when compared with the CTRL group (Table 1).

The contralateral testes did not show a significant difference in any of the variables studied, when compared to the CTRL and CO groups (Figures 1H, 3H).

In the CTRL groups and contralateral testes to CO, Leydig cells located in the central spaces of the tubular interstices were observed. In the CO group, a clear hyperplasia of this cell type was observed, interspersed between collagen fibers (Figures 4B,E). Leydig cells area were smaller compared to the other groups (Figures 4B,E insert, H).

### Stage 3: Senile

In senile CTRL animals the seminiferous tubules presented complete spermatogenesis, slight thickening of the basement membrane, and evident cell proliferation (Figure 3C). However, 33.3% of the specimens (2/6) showed damaged histological features characterized by arrest of spermatogenesis, degenerating cells, thickening and folding of the basement membrane and abundant cell desquamation (Figures 1C insert, 2C) without the presence of gonocytes.

In the senile CO group, the alterations were similar to those observed in young adult CO animals, with arrest of spermatogenesis in spermatocyte stage and the presence of atypical cells (Figure 1F). In 16.6% of the animals (1/6), electron microscopy confirmed the presence of cells with persistent gonocyte morphology (Figure 2F) and in 16.6% (1/6), Sertoli-cell-only syndrome was appreciated. When compared with the CTRL group, a significant reduction in the seminiferous epithelium area of 4.30 times, MI of 3.13 times and proliferation of 2.81 times (Figure 3F) was determined. Contrarily, the HI registered a significant increase of 4.88 times (Table 1) while lipid peroxidation did not show significant difference (*p* > 0.05) (Table 1). Testosterone concentrations showed a significant reduction of 6.51 times with respect to the CTRL group. The estradiol, LH, and FSH did not present a significant difference (*p* > 0.05) (Table 2).

In terms of the contralateral testes to the retained one in the CO group (Figure 1I), there was a significant reduction in the area of the seminiferous epithelium of 3.16 times with respect to the CTRL group. On the other hand, the HI increased by 3.90 times with respect to the CTRL group. Histological changes consisted of germ cell hypoplasia, folding and thickening of the basement membrane, some tubes with arrested spermatogenesis in spermatocytes, and other sections of tubules with complete spermatogenesis. Moreover, a significant increase in MI of 2.45 times was appreciated when compared to the CO testes. The rest of the variables were found in a position between the CTRL and CO groups with no significant difference (*p* > 0.05, Figure 3I).

In all groups, the Leydig cells remained in the same location (Figure 4). However, in the CO group, the cells were observed with hyperplasia and smaller. In the cases where the seminiferous tubules showed clear atrophy, their Leydig cells were observed to be degenerated or absent (Figures 4C,F insert,I), interspersed between a large number of collagen fibers (Figures 4B,E insert).

**Figure 5:**
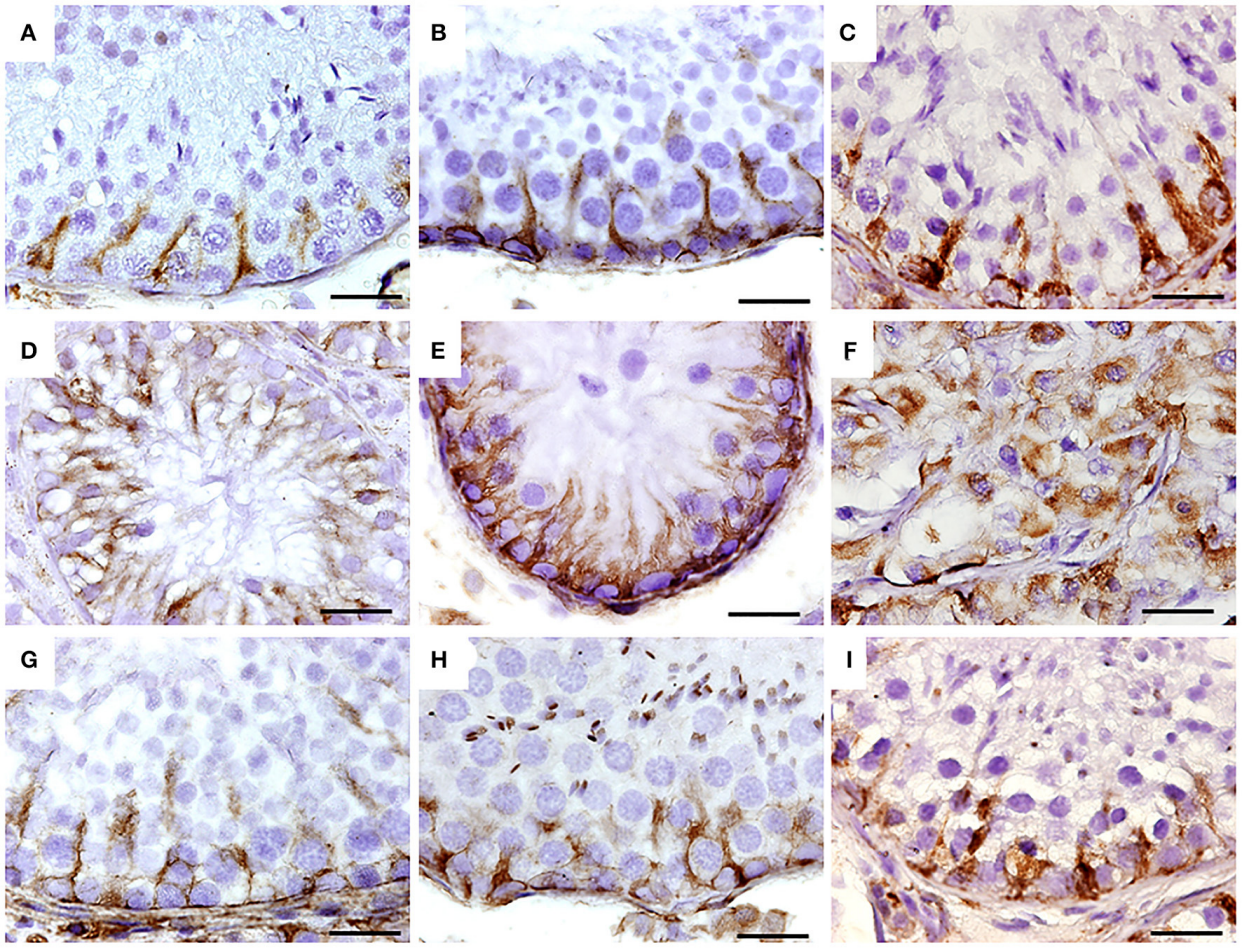
Immunohistochemistry for vimentin in seminiferous tubes of dogs from the control group (CTRL) **(A–C)**; from cryptorchidism (CO) **(D–F)** and contralateral (CONTRA) **(G–I)** in the peripubertal stages (first column); young adult (second column) and senile (third column). A marked immunoreactivity is observed in all stages of development with variation in its distribution, mainly in animals with CO. Bar scale: 50 μm.

In all stages of development, vimentin immunoreactivity was present in the cytoplasm of Sertoli cells, mainly perinuclear and in the basal compartment, forming linear columns toward the tubular lumen in the control group. In the CO group, the vimentin immunoreactivity was more evident, due to the absence of germ cells, showing the cytoplasmic extensions of the free Sertoli cells all along the tubular lumen. In the senile stage, organization of Sertoli cells was compromised. In the contralateral testicles, its distribution was similar to the control group (Figure 5).

## Discussion

Cryptorchidism occurs in dogs with high frequency (6.8%) (23) and shows anatomical and physiological similarities, as well as risk of infertility and development of neoplasia as in humans (26, 34). Therefore, this species can be considered as a suitable study model of CO.

In this study, the first seminiferous epithelium alterations in the dogs with CO appeared in the peripubertal stage. These alterations were decreased MI and delay in gonocyte differentiation to spermatogonia, evidenced by the presence of gonocytes in 50% of the specimens (3/6). These alterations generated a decrease in the number of germ cells. It has been reported in children with CO that the first histological alterations of the seminiferous epithelium begin, in subtle form, at 18 months of age with cell desquamation, folding of the basement membrane, vacuolization, lack of gonocyte differentiation to spermatogonia, with germ cell hypoplasia from the third year (35–37). Thus, at peripubertal stage, these alterations become evident. However, in this work, we did not include animals younger than 6 months of age. Hence, we were unable to determine the precise moment in which the first histological lesions appear in the pre-peripubertal stage.

In the young adult stage, alterations to the seminiferous epithelium generated by CO were evident, which was in accordance with what was reported for dogs (38, 39). These findings also coincide with what have been reported for seminiferous epithelium in humans where the thickening of the basement membrane was found (40, 41). In addition, various alterations such as germinal hypoplasia, vacuolization, desquamation, cell degeneration, presence of atypical cells, arrest in germ cell maturation (40, 42), reduction in cell proliferation (43), Sertoli-cell-only syndrome (40, 41) leading to the cessation of spermatogenesis with reduction in the MI and epithelial area have been found in humans with CO.

In our study, the presence of CO testes with Sertoli-cell-only syndrome in 22.2% of the dogs (4/18) support that this syndrome is particularly characteristic for men with a history of cryptorchidism (44). The presence of Sertoli-cell-only syndrome has also been reported in 7/10 dogs with CO in young adult animals (39), which is consistent with our results with 3/6 animals with CO in young adult status with this syndrome.

The arrest of spermatogenesis with a reduction in the area of the seminiferous epithelium, as well as in MI and cell proliferation with marked histological and ultrastructural alterations can be related to heat stress, which is known to affect the functioning of Sertoli cells in different species. The heat stress leads to downregulation in the expression of proteins of tight junctions (11, 45); thus, causing failures in spermatogenesis and cell desquamation (11, 45) as was observed in our study. Apoptotic pathways in germ cells can also be activated through redistribution of Bax protein from the cytoplasm to perinuclear of germ cells (46) and expression of the p53 mRNA (47). In this work, we observed in the CO group that spermatogenesis is arrested in spermatocytes. This is consistent with what was reported by Hirano et al. (48) who showed that pachytene spermatocytes are the most sensitive cells to temperature increases, generating DNA double-strand breaks and asynapsis, which leads to discontinuation of meiosis. Consequently, the cells are eliminated by apoptosis.

The effect of heat stress is likely due to oxidative stress generated by the production of superoxide anion, hydroxyl radical, nitric oxide and hydrogen peroxide (49–52). This phenomenon can be determined by increased malondialdehyde, the final product of lipid peroxidation (53) and which leads to the degeneration of germ cells (13). The increase in this compound observed in our animals with CO has also been seen in children with CO (54, 55). Oxidative stress in the CO patient has been shown to participate in germ cell damage (54, 55). Moreover, oxidative stress can be generated by the inactivation of the antioxidant enzymes Cu/Zn superoxide dismutase (SOD) and catalase, demonstrated in animal models (56, 57). Low SOD enzyme activity has also been found in dogs with CO (58).

These alterations are possibly owing to the fact that spermatocytes and spermatids are the germ cells most vulnerable to damage caused by reactive oxygen species because, even though they are capable of converting super oxide anion to hydrogen peroxide, they have difficulty metabolizing peroxide, which saturates its protective system against peroxide that becomes a highly toxic hydroxyl radical (59) when compared to spermatogonia or Sertoli cells. This coincides with what was observed in this work.

From young adult to senile development, CO determined histological alterations remained constant, well above the CTRL group, despite the fact that 33.3% of the CTRL animals (2/6) in this study also were experiencing alterations. None of the control animals showed testicular tumor development. There is controversy about the development of testicular tumor in senile dogs. Some authors mentioned a correlation with tumor development (60) while others assumed a contrary position (61). Seminiferous tubes without germ cells are reported in humans with unilateral and bilateral undescended testicle until old age (62) who, in some cases, did not develop testicular tumor (62).

The presence of cells with morphological and ultrastructural characteristics of gonocytes in 16.6% of the animals (2/12) in the CO dogs in the young and senile stages is uncommon. Gonocytes are not expected to be present in these stages of development; although the presence of gonocytes has been reported in 2/10 young dogs with CO (39). Hence, this event may suggest that gonocytes predisposes the development of testicular tumor (9, 15). It has been reported that in dogs with CO, the main types of testicular tumors that can develop are seminoma and mixed germ cell-sex cord stromal cell tumor, both with germ cell components, and to a lesser extent Sertoli cell tumor and interstitial cell tumor (26).

This opinion is concurrent with the proposition that the failure in the differentiation of the gonocyte to spermatogonia may be the cause of the precursor lesion of GCNIS (9, 15, 19, 63) reported that 5.4% of patients human with CO have germ cells with immunohistochemical characteristics of gonocytes.

Regarding the endocrine behavior of the patient with CO, some authors mentioned a decrease in testosterone concentrations (2, 64), while others found it unchanged (65, 66).

This reduction in testosterone concentrations in boys with cryptorchidism suggests a malfunction in the Leydig cells or the hypothalamic-pituitary-gonadal axis (67–69). Such event may alter the period of “minipuberty,” in which some authors indicate that gonocyte differentiation takes place (19, 70). However, the role of gonadotropins and testicular hormones in this process remains to be clarified (70). In CO dogs, low concentrations of testosterone have been reported from prepubertal to senile stages (71–73), as it was observed in this work, which leads to altered spermatogenesis. Leydig cell hyperplasia in the animals of CO group has been previously described in dogs with CO (30) and coincides with what has been reported for humans with this condition (74). Controverselly, in the current study we observed that the Leydig cells presented a smaller size and signs of degeneration despite being present in large numbers. A reduction in Leydig cell function has been shown in CO testes in rats (75) and humans (76), probably due to depletion of the smooth endoplasmic reticulum as it was described in humans (74).

In humans, 6.33% of patients with unilateral CO present contralateral damage, which was attributed to the impact of this pathology on both gonads (38, 77). In the contralateral testes, the reduction in the area of the seminiferous epithelium when compared with CTRL group and the increase in MI when compared to the CO group suggest that the contralateral position conditions testicular damage. The level of damage places the contralateral testes between the CTRL and CO groups as reported by Kawakami et al. (78) in dogs. However, some authors mentioned that this may be caused by deficient cooling system in the venous and arterial circulation between both gonads, thus, constant heat stress is maintained (75). In any case, in this study, no difference in lipid peroxidation was observed when compared to the CTRL and CO groups. Hence, we suggest that the mechanism of damage may be through another pathway such as Sertoli cell dysfunction determined by low concentrations of transferrin, which has been associated with low concentrations of testosterone, and which has been reported in the contralateral testis of dogs with surgically induced CO (78).

Greater intensity and disorganization of vimentin immunoreactivity have been reported in rats and humans with CO (79–81) and in animals exposed to oxidative stress (81, 82). Although Pecile et al. (83) do not report changes in vimentin immunoreactivity in the testes of dogs with CO, their images show a loss in the linear organization of immunoreactivity to this intermediate filament (83). Damage and restoration of spermatogenesis are related to the disintegration or recovery of these filaments (84).

The results of this work demonstrate that the testicular development of the dog with CO is similar to that of the human with this pathology, provoking alterations of the seminiferous epithelium that lead to problems in fertility and possible risk of developing neoplasia. Therefore, we suggest that this species could be used as a suitable model for the study of CO. This may allow research for compounds that would reduce fertility problems and the risk of developing testicular tumor, as well as provide early risk markers for the development of GCNIS in patients with CO.

## Data availability statement

The raw data supporting the conclusions of this article will be made available by the authors, without undue reservation.

## Ethics statement

The animal study was reviewed and approved by INP: 54/2020. Written informed consent was obtained from the owners for the participation of their animals in this study.

## Author contributions

Experiments were conceived and designed and the paper was written by RMV-V, JCR-C, and NH-J. Experiments were performed by NH-J, ER-C, and FR-D. Data were analyzed by RMV-V, NH-J, JCR-C, DL-H, MJ-M, RR-R, and AM. All authors contributed to the article and approved the submitted version.

## Funding

This work was supported by Instituto Nacional de Pediatria (Grant Number INP: 54/2020).

## Conflict of interest

The authors declare that the research was conducted in the absence of any commercial or financial relationships that could be construed as a potential conflict of interest.

## Publisher's note

All claims expressed in this article are solely those of the authors and do not necessarily represent those of their affiliated organizations, or those of the publisher, the editors and the reviewers. Any product that may be evaluated in this article, or claim that may be made by its manufacturer, is not guaranteed or endorsed by the publisher.
